# Larval density mediates knockdown resistance to pyrethroid insecticides in adult *Aedes aegypti*

**DOI:** 10.1186/s13071-018-2865-x

**Published:** 2018-05-03

**Authors:** Marissa K. Grossman, Valentin Uc-Puc, Adriana E. Flores, Pablo C. Manrique-Saide, Gonzalo M. Vazquez-Prokopec

**Affiliations:** 1 Department of Entomology, Pennsylvania State University, University Park, Pennsylvania, PA 16802 USA; 2 Departamento de Zoología, Campus de Ciencias Biológicas y Agropecuarias, Universidad Autónoma de Yucatán, Apartado Postal 4-116, Itzimna, 97000 Mérida, Yucatan Mexico; 3 Universidad Autónoma de Nuevo León, Facultad de Ciencias Biológicas, Av. Universidad s/n Cd. Universitaria, San Nicolas de los Garza, 66455 Nuevo Leon, Mexico; 4 Department of Environmental Sciences, Emory University, 400 Dowman Drive, Atlanta, GA 30322 USA

**Keywords:** Pyrethroid resistance, *Aedes aegypti*, Intraspecific competition, *kdr*

## Abstract

**Background:**

Understanding mechanisms driving insecticide resistance in vector populations remains a public health priority. To date, most research has focused on the genetic mechanisms underpinning resistance, yet it is unclear what role environmental drivers may play in shaping phenotypic expression. One of the key environmental drivers of *Aedes aegypti* mosquito population dynamics is resource-driven intraspecific competition at the larval stage. We experimentally investigated the role of density-dependent larval competition in mediating resistance evolution in *Ae. aegypti*, using knockdown resistance (*kdr*) as a marker of genotypic resistance and CDC bottle bioassays to determine phenotype. We reared first-instar larvae from susceptible and pyrethroid-resistant field-derived populations of *Ae. aegypti* at high and low density and measured the resulting phenotypic resistance and population *kdr* allele frequencies.

**Results:**

At low density, only 48.2% of the resistant population was knocked down, yet at high density, the population was no longer phenotypically resistant - 93% were knocked down when exposed to permethrin, which is considered susceptible according to WHO guidelines. Furthermore, the frequency of the C1534 *kdr* allele in the resistant population at high density decreased from 0.98 ± 0.04 to 0.69 ± 0.04 in only one generation of selection.

**Conclusions:**

Our results indicate that larval conditions, specifically density, can impact both phenotype and genotype of pyrethroid-resistant populations. Furthermore, phenotypic susceptibility to pyrethroids may be re-established in a resistant population through a gene x environment interaction, a finding that can lead to the development of novel resistance management strategies that capitalize on density-induced costs.

## Background

Insecticide resistance poses a significant threat to the control of both agricultural pests and vectors of human disease. As insecticides remain one of the pillars of contemporary vector-borne disease control, curtailing resistance evolution or delaying undesirable impacts of resistance on pathogen transmission are global health priorities [1]. Current research on insecticide resistance in disease vectors has mainly focused on its evolutionary underpinnings: the genes responsible, the physiologic pathways involved, and how different control methods can potentially delay the evolution of resistance [1–4]. While genetic mechanisms have been largely identified, it is still unclear what role environmental drivers play in shaping phenotypic expression. For example, it has been shown that rearing *Anopheles* spp. under reduced diet regimes significantly decreases their phenotypic resistance to permethrin [5] and DDT [6], and adult insecticide exposure at lower temperatures increases their phenotypic resistance to malathion [7]. Understanding the potential for gene × environment interactions to impact phenotype could lead to novel approaches for resistance management and mitigation.

Using *Aedes aegypti* mosquitoes as a study system, we aim to explore the impact of intraspecific competition at the larval stage on the genotype and phenotypic expression of pyrethroid resistance. *Aedes aegypti*, the main vector for dengue, chikungunya and Zika, experiences strong insecticide selection pressure from vector control efforts that are currently the only way to prevent disease outbreaks. Most *Ae. aegypti* control programs throughout the world employ ultra-low volume spraying (ULV), indoor residual spraying (IRS), and the application of larvicides to target individuals in both the aquatic and terrestrial life stages [8, 9]. Consequently, *Ae. aegypti* has developed resistance to every class of insecticide, with the most widespread being pyrethroid resistance [10]. The most common mechanism conferring resistance to synthetic neurotoxins such as pyrethroids is called “knockdown resistance” or *kdr*. These are point mutations in the para*-*orthologous sodium channel gene that alter the ability of the insecticide to bind to the voltage-gated sodium channels in the mosquito’s nerve cell membranes [11]. Given the critical role of voltage-gated sodium channels in nervous system functioning, the presence of *kdr* mutations within a mosquito’s genome has important pleiotropic effects on mosquito behavior, performance and overall fitness [12–14].

It has been well established that density-dependent growth at the larval stage is one of the main exogenous factors shaping *Ae. aegypti* population dynamics [15–18]. In natural conditions, the *Ae. aegypti* life-cycle, which involves four free-living aquatic larval stages and a pupal stage that does not feed, occurs mainly in man-made containers such as buckets or flower pots [19]. During the larval period, strong indirect competition for resources occurs, particularly in larval habitats with limited food availability [15, 16, 20]. At high larval density, per-capita consumption is reduced, leading to smaller adult mosquitoes, slower development time, and decreased adult survival [15–17]. What is yet unknown is whether intraspecific competition in the larval stage can have a differential impact on the performance of insecticide-resistant or susceptible individuals, thereby influencing resistance evolution. For example, Raymond et al. [21] found that under high density conditions, a Cry1Ac (*Bacillus thuringiensis* toxin) resistant population of diamondback moth (*Plutella xylostella*) had reduced survival compared to the susceptible population, and the resistant population also experienced a significant decline in phenotypic resistance in only three generations.

The strong bottom-up population regulation exerted by larval intraspecific competition in *Ae. aegypti* led us to hypothesize that density-dependent competition can significantly influence the allelic frequency of the *kdr* mutations in a population and influence the rates of resistance evolution. To test this hypothesis, we designed a series of larval competition experiments to investigate the role of density-dependence at the larval stage on the rates of genotypic and phenotypic resistance to pyrethroid insecticides.

## Methods

### Experimental design

To quantify the impact of larval intraspecific competition on the resulting insecticide resistance status, we created a fully factorial experiment with two factors: density and population. There were two levels of density, low (50 larvae) and high (500 larvae), which represent the lower and upper range of larval density described in Merida, Mexico during the dengue transmission season [22, 23]. We used F1 larvae from two *Ae. aegypti* field populations: susceptible (10% frequency of both *kdr* mutations and 100% knockdown to permethrin at the diagnostic time) and resistant (98% frequency of the C1534 mutation, 73% of the I1016 mutation and 13% knockdown to permethrin at the diagnostic time). We then mixed the two populations as first-instar larvae (see below for details) to create a third, intermediate resistance level. Our experimental design thus involved six treatment combinations (2 densities and 3 resistance levels), which were replicated five times each.

We used 2 l white experimental buckets, which is the typical size habitat for *Ae. aegypti* in Merida, and filled each one with 1 l of municipal water [23]. We placed either 50 (low density treatment) or 500 (high density treatment) first-instar larvae from each population (susceptible or resistant) into an experimental bucket. The intermediate resistance level was created by placing 25 or 250 first-instar larvae from each resistance population into the same experimental bucket. Larvae were fed 50 mg of bovine liver powder (MP Biomedicals LLC, Santa Ana, USA) every other day until all reached pupation. Buckets were covered with a mesh net to protect from oviposition of ambient mosquitoes and entrance of other organisms. The number of pupae and recently emerged adults in each bucket were counted daily. Adults were removed daily with a mouth aspirator and placed in an experimental cage (BugDorm-1 Insect Rearing Cage, MegaView Science, Talchung, Taiwan) and given 75–100 ml of 5–10% sugar solution every day for hydration and nourishment.

Once all adult mosquitoes emerged in the low density treatment, 15 females and 15 males from each replicate were selected at random, euthanized by freezing, and stored individually in 100% ethanol for further genotyping analysis. For the high density treatment, the same procedures were conducted; however, half of the 30 mosquitoes (7 males and 7 females) were removed halfway through the experiment (day 18), and the other half were removed at the end. Obtaining two samples over the duration of the high density experiment minimized any potential bias if emergence time differed between susceptible and resistant individuals.

Experiments were conducted in Merida, Mexico during February-May 2016, inside an urban residence to recreate the typical environmental fluctuations experienced by *Ae. aegypti* populations. Temperature ranged from 21.9 to 37.1 °C and humidity from 40 to 82% throughout the course of the experiment, measured inside the experimental room with a RadioShack® Indoor/Outdoor Thermometer (RadioShack, Fort Worth, USA).

### Phenotypic resistance assay

Standardized Centers for Disease Control and Prevention (CDC) bottle bioassays were conducted on mosquitoes from each treatment replicate to determine phenotypic resistance. Four replicates of 25 mosquitoes each were placed in bottles coated with 15 μg/ml of technical grade permethrin according to CDC guidelines [24]. In the high density treatment, two of the four replicates were conducted at day 18, using mosquitoes that had emerged prior to that date (3–4 days-old), so as to not bias results as previously stated; the other two replicates were completed at the end of the experiment, which was between days 33–40 depending on the replicate. The low density treatment did not contain enough mosquitoes for the bottle bioassays since they only contained 50 mosquitoes at maximum, so two extra replicate buckets were simultaneously run but only used to complete the bioassays. The number of individuals knocked down was recorded every 10 minutes until all individuals were knocked down, but no longer than 120 minutes. The percentage of individuals knocked down at the diagnostic time of 30 minutes was calculated for each replicate, and phenotypic resistance was defined as this percentage.

### Genotype analysis

DNA was extracted from 30 individuals per replicate using the salt extraction method [25], and then tested with allele-specific real time PCR to determine genotype at the 1534 and 1016 loci following protocols described in Alvarez et al. [26].

### Statistical analyses

Allele frequencies for C1534 and I1016 were calculated for each population before and after the experiment. To test for linkage disequilibrium between the two markers, we calculated the coefficient D and the resulting r^2^ following the equations outlined in Gillespie [27] and used a Chi-square test with one degree of freedom to test the statistical significance. The change in genotype was analyzed with a Chi-square test of independence, and the difference between densities in the proportion knocked down with insecticide was assessed with a Welch t-test. Differences in development time between high and low density, defined as the total number of days from first-instar larva to adult, was quantified using a linear mixed effects model with replicate as a random intercept (R package *nlme* [28]). The probability of survival was analyzed using a binomial-distributed generalized linear mixed effects model (GLMM), also with replicate as a random intercept (R package *lme4* [29]). Analyses were conducted with the R statistical program version 3.3.2 [30].

### *Aedes aegypti* strain description

Pyrethroid susceptible and resistant *Ae. aegypti* field strains were generated from eggs collected in the cities of Cienega de Flores (Nuevo Leon State, Mexico, susceptible strain) and Uman (Yucatan State, Mexico, resistant strain). First generation (F1) mosquito larvae were used for the experiments. Initial gene frequencies for each colony for the C1534 and I1016 *kdr* mutations were quantified using allele-specific real time PCR on 50 randomly selected F0 adult mosquitoes, applying the protocols described in Alvarez et al. [26]. The susceptible population had a 10% frequency of both *kdr* mutations and 100% knockdown to permethrin at the diagnostic time, and the resistant population had a 98% frequency of the C1534 mutation, 73% of the I1016 mutation and 13% knockdown to permethrin at the diagnostic time. Additional information on the resistant and susceptible strains can be found in Deming et al. [22] and Siller et al. [31], respectively. Rates of phenotypic resistance to the pyrethroid permethrin were estimated using the standardized CDC bottle bioassays following published guidelines on 100 F1 mosquitoes of each colony [24].

We acknowledge that our experimental strains are derived from different locations and therefore do not share the same genetic background, though we are only using the susceptible strain as a control. The statistical analysis conducted does not compare the strains; rather, it compares each strain to itself given density treatment (high and low) since that is our main question of interest. Including the susceptible strain allows us to rule out other environmental conditions that could be causing changes in resistance status.

## Results

### Larval development

**Fig. 1:**
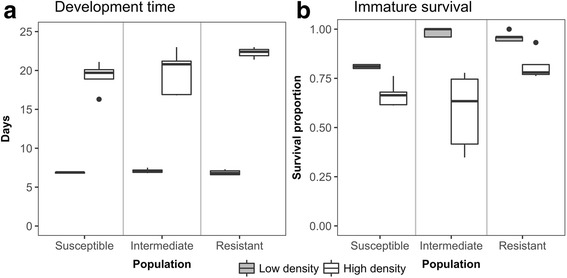
Larval performance for each population and density. Boxplots show the distribution of **a** development time, defined as total time from first-instar to adult and **b** immature survival, measured as the proportion of the population surviving to adult of all five replicates

The mean (± standard deviation) number of days from first-instar larva to adult was 12.3 ± 0.6 days longer at high density than low density (Fig. 1a; GLMM generalized linear mixed-effects model, *t* = 21.93, *df*= 5856, *P* < 0.0001). Similarly, the survival probability from first-instar larva to adult was significantly lower for individuals in the high density treatment than the low density (Fig. 1b; Binomial GLMM, odds ratio OR = 0.15; 95% CI: 0.11–0.20).

### Phenotypic resistance

**Fig. 2:**
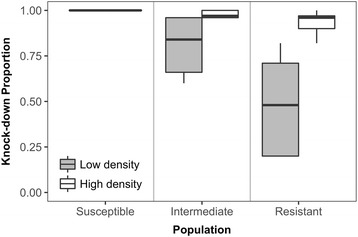
Phenotypic resistance changes based on density conditions. Boxplots show the distribution of the proportion of the population knocked down in the presence of the diagnostic dose of permethrin according to CDC bottle bioassay standard procedures

Expression of phenotypic resistance to the pyrethroid insecticide permethrin was significantly lower in the resistant population at high density compared to low density (Fig. 2; Welch t-test, *t* = -3.41, *df* = 4.5, *P* = 0.0225). At low density, only an average of 48.2 ± 28.5% of the resistant population was knocked down at 30 minutes, but at high density, an average of 93 ± 7.1% were knocked down. This significant reduction in phenotypic resistance at high density rendered the originally “resistant” population susceptible according to the WHO guidelines, which mark the resistance threshold at 90% population knockdown at the diagnostic time for the CDC bioassays [32]. An increase in susceptibility at high density was also seen in the intermediate population, with an average 80.4 ± 16.7% of the population knocked down at low density, yet an average of 97.8 ± 2.0% knocked down at high density (Fig. 2).

### *kdr* allele frequencies

**Fig. 3:**
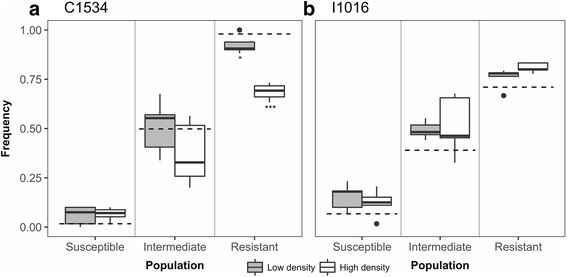
*kdr* frequencies as a result of density-dependent selection. Box plots show the distribution of the *kdr* frequencies of adult mosquitoes emerging from each treatment combination. The dotted line represents the initial frequency of first-instar larvae in the population. *P < 0.01, ***P < 0.0001

At both densities, the C1534 allele frequency of the resistant population was significantly reduced from a starting overall frequency of 0.98 to an average ± SD frequency of 0.93 ± 0.05 at low density (Fisher’s exact test, *P* = 0.003) and 0.69 ± 0.04 at high density (Fisher’s exact test, *P* < 0.0001) (Fig. 3). This marked effect of density on allele frequency change was not observed for the I1016 mutation (Fig. 3). The frequency of the I1016 allele increased slightly after both treatments, though not significantly (low density: Chi-square = 0.47, *P* = 0.492; high density: Chi-square = 3.3, *P* = 0.068).

## Discussion

We found that density-dependent intraspecific competition can act as a selective force to regain susceptibility in pyrethroid-resistant *Ae. aegypti* mosquitoes. High density larval conditions induced competition, evidenced through reduced immature survival and delayed development time. Consequently, this heightened competition selected for individuals without the C1534 *kdr* mutation, causing a striking decrease in its frequency in the resistant population only after one generation of selection. Such rapid evolution gives insights into the maintenance of polymorphism at *kdr* sites in *Ae. aegypti* field populations. Although insecticide selection pressures are strong, they are rarely uniform in time or space, as they are largely driven by disease outbreaks [33]. In the absence of insecticide, population densities may increase, imposing stronger competition and selection towards susceptibility. The alternation of insecticide selection pressure with selection due to density-dependence may, in part, account for the genetic variation at *kdr* loci and can be leveraged to mitigate resistance evolution.

Equally important is that phenotypic susceptibility was re-established in the resistant population through a gene-environment interaction. Larvae from the same parent population with high resistance exhibited different resistance phenotypes depending on the conditions in which they were raised. If raised with minimum intraspecific competition (low density), they remained resistant; however, if they were raised under strong competition (high density), they became diagnostically susceptible according to WHO guidelines [32]. Not only do these results demonstrate that phenotypes can be altered based on environmental conditions, but they also raise concerns about the external validity of biological assays used to phenotypically characterize the levels of resistance of natural populations. Based on our findings, we hypothesize that the standard resistance assays (CDC bottle bioassay and the WHO susceptibility test) would provide different results if performed with adults collected from the field (that are naturally constrained by food and density) *versus* adults reared in optimal laboratory conditions. This potential discrepancy could bias results from standard bioassay assays that are used to monitor resistance in field populations, providing incomplete or inaccurate information for vector control. Further evaluations of the bioassay methodology are needed, including the investigation of the correlation between phenotype and genotype at higher insecticide doses and the interplay between density and phenotypic resistance.

The impact of the larval environment on phenotypic resistance in adults has also been demonstrated in *Anopheles* spp. Owusu et al. [5] found that a nominally pyrethroid-resistant population of *An. stephensi* became susceptible according to WHO guidelines (using WHO tube tests) if reared with only 25% of the larval food available. Additionally, Oliver et al. [6] found that DDT-resistant *An. arabiensis* had decreased time to mortality using the bottle bioassay when larvae were on a restricted diet compared to well-fed larvae. Given these pervasive effects across species, it seems important to re-evaluate existing diagnostic assays for resistance as well as understand how resistance may change with varying environmental conditions.

This study investigated the effect of density-dependent competition on pyrethroid resistance in only one generation, though it would be beneficial to assess the impacts over multiple generations of density-induced selection. While we found a marked reduction in the C1534 *kdr* mutation frequency in one generation, we did not find a significant change in the I1016 mutation. This is likely because we only conducted the experiment over one generation; when Brito et al. [12] reared a population of *Ae. aegypti* with the I1016 *kdr* mutation in the absence of insecticide for 15 generations, they found a decrease in the I1016 allele from 50% to 21.7%, which would on average produce a 1.8% change in one generation. Assessing the longer-term effects of density-induced selection could aid in our understanding of both field dynamics and potential fitness costs of the *kdr* mutations.

## Conclusions

Here we show adult pyrethroid resistance can be mediated by larval rearing density. Intraspecific competition induced by high densities can render a “resistant” population susceptible and act as a selective force to reduce the C1534 *kdr* allele frequency. Given that *Ae. aegypti* are container breeders and subject to density-dependence in the field, the results give insight into one of the mechanisms driving insecticide resistance evolution in field populations. Furthermore, the results highlight the deficiencies in current diagnostic assays for resistance and suggest that future research should explore how to make these assays more robust to differences in environmental conditions.

## Abbreviations

CDC: Centers for Disease Control and Prevention
DDT: Dichlorodiphenyltrichloroethane
*kdr*: knockdown resistance
PCR: polymerase chain reaction
WHO: World Health Organization
